# High levels of dietary methionine improves sitagliptin-induced hepatotoxicity by attenuating oxidative stress in hypercholesterolemic rats

**DOI:** 10.1186/s12986-019-0422-z

**Published:** 2020-01-06

**Authors:** Avinash Kumar, Rashmi Pathak, Henry A. Palfrey, Kirsten P. Stone, Thomas W. Gettys, Subramanyam N. Murthy

**Affiliations:** 1 Environmental Toxicology Department, Southern University and A&M College, Baton Rouge, LA 70813 USA; 2 Laboratory of Nutrient Sensing and Adipocyte Signaling, Pennington Biomedical Research Center, Baton Rouge, LA USA

**Keywords:** Methionine, Hepatotoxicity, Cholesterol, Sitagliptin, Oxidative stress

## Abstract

**Background:**

Both cholesterol (Cho) and methionine (Met, a precursor for homocysteine) are risk factors for fatty liver disease. Since Western diets are rich in Cho and Met, we investigated the hepatic effects of feeding a diet enriched in Met and Cho. Further, based on the reported anti-oxidative and lipid lowering properties of sitagliptin (an antidiabetic drug), we tested whether it could counteract the negative effects of high Cho and Met. We therefore hypothesized that sitagliptin would ameliorate the development of liver pathology that is produced by feeding diets rich in either Cho, Met, or both.

**Methods:**

Male Sprague Dawley rats were fed ad libitum a) control diet, or b) high Met or c) high Cho, or d) high Met + high Cho diets for 35 days. From day 10 to 35, 50% of rats in each dietary group were gavaged with either vehicle or an aqueous suspension of sitagliptin (100 mg/kg/day). Liver samples were harvested for histological, molecular, and biochemical analyses.

**Results:**

The high Cho diet produced significant hepatic steatosis which was unaffected by sitagliptin. Contrary to expectation, sitagliptin exacerbated expression of hepatic markers of oxidative stress and fibrosis in rats fed high Cho. Corresponding increases in 4-hydroxynonenal adducts and collagen deposition were demonstrated by immunohistochemistry and sirius red staining. These hepatic changes were absent in rats on the high Met diet and they were comparable to controls. The inclusion of Met in the high Cho diet resulted in significant reduction of the hepatic steatosis, oxidative stress, and fibrosis produced by high Cho alone.

**Conclusion:**

Sitagliptin exacerbated the effects of high Cho on both oxidative stress and fibrosis, resulting in NASH like symptoms that were significantly reversed by the inclusion of Met.

## Introduction

The widespread increase in the incidences of non-alcoholic fatty liver disease (NAFLD), cardiovascular disease (CVD), diabetes, and obesity are a global health concern. Several of these conditions can be reasonably managed by lifestyle changes including diet and exercise. Animal products such as meat, poultry, and dairy are rich in cholesterol (Cho) and methionine (Met). Cho and Met are therefore consumed together when animal products are major components of the diet. NAFLD affects 15–46% of adults and is currently considered the third most common cause of cirrhosis and hepatocellular carcinoma (HCC) following hepatitis C infection and alcoholic liver diseases [1]. NAFLD is projected to become the leading cause for cirrhosis and HCC over the next 10–20 years [2]. Diabetes and obesity also play a prominent role in NAFLD and their incidences are increasing [3].

The strong association between high Cho and inflammatory diseases like NAFLD and atherosclerosis is well established [4–6]. In addition to de novo synthesis, dietary intake of Cho contributes significantly to its high circulating levels. Recent studies suggest that dysregulation of Cho homeostasis and accumulation of free Cho in hepatocytes is involved in the progression of NAFLD and non-alcoholic steatohepatitis (NASH) [7–9]. Excess dietary Cho leads to hepatic inflammation and NASH-like symptoms but not liver steatosis in hyperlipidemic mice [8]. Excess circulating levels of Cho can be lipotoxic and trigger liver injury leading to NASH by virtue of their ability to form reactive oxygen species (ROS) and increase oxidative stress. Free Cho can also induce cellular toxicity by activating Kupffer cells and hepatic stellate cells, inducing the unfolded protein response (UPR). All these effects can contribute to mitochondrial dysfunction, and amplify oxidative stress [9]. Further, increased accumulation of free Cho has also been shown to induce necrosis and apoptosis in hepatocytes [10].

Methionine (Met), a sulfur-containing essential amino acid obtained from dietary sources, is involved in protein synthesis. In addition to its roles in cellular redox function, one-carbon metabolism, and pyrimidine synthesis, Met serves an essential role in initiation of eukaryotic protein biosynthesis [11, 12]. The activated form of Met, S-adenosylmethionine (SAM) serves as a donor of methyl groups for many biological reactions. SAM, after donating its methyl group to methyl group acceptor molecules, is converted to S-adenosylhomocysteine (SAH) which is de-adenosylated to homocysteine (Hcy). This non-protein amino acid is the end product of the transmethylation cycle of Met metabolism. Since Met (mainly metabolized in the liver) is a precursor for Hcy, diets rich in Met can increase circulating levels of Hcy, resulting in hyperhomocysteinemia (HHcy). Additionally, deficiencies of vitamins B6, B12, and folate or mutations in genes of Met/Hcy metabolism can also cause HHcy. The association between HHcy and NAFLD is well documented [13]. A recent study by Yamada and coworkers demonstrated that feeding six times higher Met than a standard diet containing 0.44% Met induces hepatocyte injury in mice by altering hepatic lipid metabolism and inducing oxidative stress [14]. In another study, it was demonstrated that excess SAM triggered the synthesis of triglycerides, ultimately leading to hepatic steatosis and the development of NAFLD [15]. Additionally, it was reported that elevated levels of Hcy induce hepatic steatosis by activating ER stress responses [16]. ER stress responses in turn can result in oxidative stress, inflammatory cell infiltration and fibrosis [17]. Moreover, elevated levels of Hcy thiolactone (HTL; an intramolecular thioester of Hcy) were found in response to increasing levels of Hcy as demonstrated in studies involving human cells and animal models [18, 19]. Importantly, HTL has been shown to induce oxidative stress [20, 21] which has been implicated in several pathologies associated with HHcy [22].

Met has been considered to be the most toxic amino acid based on the studies comparing the relative toxicities of ingested amino acids [23, 24]. Russell et al. compared the effects of same percentage excess of essential amino acids on growth rate by individually adding twice the requirement of each amino acid to diet and found that excess Met resulted in maximum growth retarding effect in rats [25]. In contrast, reduction in intake of Met or Met restriction is reported to result in beneficial effects like increase in energy expenditure, longevity, improvement of insulin sensitivity and reduction in adiposity [26–28]. There are animal studies documenting toxic effects of diets supplemented with high levels of Met (2% and above) [14, 16]. Importantly, Met has been reported to induce hypercholesterolemia (HChol) by enhancing Cho synthesis in the liver [29].

However, there are also studies which suggest that Met directly or its intermediate SAM have protective effects particularly in cases of drug overdose [30, 31]. Neuvonen and coworkers demonstrated that Met when added to paracetamol reduced acute toxicity of paracetamol by 50% in rats [30]. In a related study, intraperitoneal injection of SAM protected against acetaminophen-induced liver injury in mice primarily by preventing glutathione depletion and mitochondrial dysfunction [31]. Several intermediates formed during the metabolism of Met have also been shown to have anti-oxidative properties [32].

The combined effects of high Cho and high Met in the context of hepatic oxidative stress and fibrosis are not adequately studied. Since HChol and HHcy have been individually shown to result in hepatic lipid accumulation, inflammation and oxidative stress, we investigated the combined effects of feeding high Cho and high Met in inducing hepatic oxidative stress and fibrotic responses. Importantly, in anticipation of seeing robust increase in hepatic oxidative stress, fibrosis, and lipid accumulation in rats fed a combination of high Cho with high Met, we investigated the role of sitagliptin in alleviating these effects. Sitagliptin, an antidiabetic drug belonging to the class of dipeptidyl peptidase-4 (DPP-4) inhibitors, reduces post-prandial glycemia via inhibition of the degradation of glucagon like peptide-1 (GLP-1). In addition to their antidiabetic action, DPP-4 inhibitors have several other health benefits such as improving lipid profile, reducing inflammation, oxidative stress, hepatosteatosis and insulin resistance [33–35]. Therefore, 35 day long studies were conducted using male Sprague Dawley rats that were fed a dietary excess of Cho and/or Met, and administered with sitagliptin. The effects of these diets and drug on hepatic oxidative stress and fibrosis are reported currently. A key finding of this study was the increase in oxidative stress and fibrosis by sitagliptin in rats on the high Cho diet, and its attenuation by the addition of Met.

## Materials and methods

### Animal experiments

#### Experiment-1

The approval for all experiments was obtained from the institutional animal care and use committee (IACUC) of Pennington Biomedical Research Center. Male Sprague Dawley (SD) rats between 250 and 270 g were obtained from Envigo RMS, Inc. (Indianapolis, IN). The rats were weight-matched and divided into 4 groups (*n* = 7) and fed Control (Con), high Methionine (Met), high Cholesterol (Cho), or Methionine+Cholesterol (MetCho) -enriched diets. Purina rodent diet (#5001) with 0.5% cholic acid and 2% maltose dextrin served as the Con diet. The high Met diet was made by enriching the Con diet with 1.5% methionine, the high Cho diet by enriching the Con diet with 2% cholesterol, and the high Met+Cho diet by enriching the Con diet with 1.5% methionine + 2% cholesterol. The energy content of Con, Met, Cho and MetCho diets were 12.71 kJ/g, 12.77 kJ/g, 12.46 kJ/g and 12.52 kJ/g, respectively. All rats were provided their respective diets ad-libitum, with free access to water. The experiment lasted for 35 d and the rats were maintained in a light-controlled room (12:12 h day/night cycle) under a constant temperature (22 °C). Rats were housed in cages with standard bedding.

#### Experiment-2

Weight-matched adult male SD rats were divided into 4 groups (*n* = 7) and fed the Con diet, the Met diet, the Cho diet, or the MetCho diet for a period of 35 days. From day 10 through day 35, all rats were administered an aqueous solution of sitagliptin (100 mg/kg body weight/day) by oral gavage. In this experiment we had an additional Con group and rats in this group were gavaged with vehicle (water); vehicle Con group. Fasting blood glucose was measured at weekly intervals using a glucometer and tail vein sampling. As was done in the first experiment, an initial blood sample was collected before starting the dietary regimen and a final blood sample was obtained at the end of the experiment.

#### Experiment-3

For experiment 3, weight-matched adult male SD rats were randomly assigned to 3 groups (*n* = 16 per dietary group) and fed the Con diet or diets enriched with Cho or with MetCho. From each group 50% of rats (*n* = 8) were gavaged with an aqueous solution of sitagliptin (100 mg/kg body weight/day) while the remaining 50% were gavaged with vehicle (water) orally starting from day 10 through day 35. Fasting blood glucose was measured at weekly intervals using a glucometer and tail vein sampling.

### Measurement of body composition

Body weight and body composition of all rats were measured at weekly intervals for the duration of the experiments. Body composition was measured using time domain-NMR spectroscopy (Bruker Minispec, Billerica, MA). The instrument was calibrated using the appropriate standards for fat, lean mass and water as per the protocol of the manufacturer.

### Sample collection

The final blood sample was collected at the end of each experiment by cardiac puncture (after CO_2_ inhalation just before euthanasia). Serum was separated by centrifugation and stored at −80°C for the analysis of biochemical parameters. All the lobes of the liver were carefully dissected and a small segment from the largest lobe of the liver was processed for fixation, paraffin embedding, and sectioning for histological analysis. The remaining tissue was snap frozen in liquid nitrogen and stored at −80°C for further analysis.

### RNA isolation and real time PCR

 RNA was isolated from liver using TRIzol (MRC, Inc., Cincinnati, OH) and homogenized by a hand-held homogenizer. After incubation for 5 min at room temperature, 1-bromo-3-chloropropane (Sigma-Aldrich, St. Louis, MO) was added and vortexed. After centrifugation at 12,000 rpm for 15 min at 4^o^C, the supernatant was transferred to a fresh tube for the addition of 70% ethanol (1:1). Total RNA was isolated using RNeasy mini kit (Qiagen, Germantown, MD) according to the manufacturer’s protocol and RNA samples were quantified on a NanoDrop spectrophotometer (Thermo Fischer Scientific, Waltham, MA). 2.0 μg of total RNA was reverse-transcribed using Oligo-(dT)20 primers and M-MLV reverse transcriptase using the kit from Promega (Madison, WI). 10 ng of cDNA was used for quantitative real-time PCR on a Step One Plus System (Applied Biosystems, Foster City, CA). The sequences of primers used in this study are provided in Table 1. Target gene expression in each sample was normalized to the endogenous control gene cyclophilin in each sample.

**Table 1:** Sequence of primers used for quantitative real-time PCR

Target gene	Sequence
*Cypa*	
Forward	TATCTGCACTGCCAAGACTGAGTG
Reverse	CTTCTTGCTGGTCTTGCCATTCC
*Nox2*	
Forward	CTCTGCCTCCATTCTCAAGTC
Reverse	GCGAACCACTCAAAAGCATG
*Lox1*	
Forward	CCCACAAGTCACAGACTCTTC
Reverse	CACACACTCACACACACAAATAC
*Inos*	
Forward	GGAGCAGGTTGAGGATTACTTC
Reverse	TCAGAGTCTTGTGCCTTTGG
*Cd36*	
Forward	GGCGATGAGAAAGCAGAAATG
Reverse	CACTACTCCAACACCAAGTAAGA
*Klf2*	
Forward	ACTTGCAGCTACACCAACTG
Reverse	CTGTGACCCGTGTGCTTG
*Tgfb*	
Forward	AGAGCCCTGGATACCAACTA
Reverse	CAACCCAGGTCCTTCCTAAAG
*Tlr4*	
Forward	GGAAAAGCCTTGAATCCAGATG
Reverse	AGCAGAAACCCAGATGAACTG
*Tlr2*	
Forward	ATGAACACTAAGACATACCTGGAG
Reverse	CAAGACAGAAACAGGGTGGAG
*a-Sma*	
Forward	GCTCCTCCAGAACGCAAATA
Reverse	CAGCTTCGTCATACTCCTGTTT
*Mmp9*	
Forward	CTTGAAGTCTCAGAAGGTGGATC
Reverse	CGCCAGAAGTATTTGTCATGG
*Timp1*	
Forward	CCACCTTATACCAGCGTTATGAG
Reverse	GGTTCTGGGACTTGTGGAC

### Histological evaluation by H&E staining

The liver samples were fixed in 10% Neutral Buffer Formalin and processed on a TissueTek VIP 6 Vacuum Infiltration Processor. They were embedded in paraffin and 5 μm sections were obtained for staining with hematoxylin and eosin (H&E). The H&E staining was performed using a Leica St 5020 Autostainer (Buffalo Grove, IL) and the slides were used for microscopy and histopathological examination. Also, the sections were scanned at 20X using a Hamamatsu Nanozoomer Digital Pathology system (Hamamatsu City, Japan).

### Immunohistochemistry

Immunohistochemistry was performed on paraffin embedded liver sections. Briefly, the slides were first deparaffinized using xylene and dehydrated using ethanol. Theses slides were then pressure heated at 100 °C for 20 min in Na-citrate buffer. To inactivate the endogenous peroxide activity slides were kept for 12 min at room temperature in 3% H_2_O_2_ in TBS. Further, the slides were incubated with blocking buffer for 30 min to block non-specific binding sites followed by overnight incubation in anti 4-HNE primary antibody (Abcam). Detection was performed using Leica Bond Polymer Refine kit and slides were counterstained with hematoxylin. The stained slides were scanned using Hamamatsu Nanozoomer Digital Pathology system (Hamamatsu City, Japan) and the digital information were stored for analysis.

### Measurement of liver triglycerides

The triglyceride content in the liver tissue was assayed using a commercially available kit from Cayman chemicals (Ann Arbor, MI). Briefly, 40–50 mg of liver tissue was homogenized in the diluted NP40 buffer containing protease inhibitor cocktail. The homogenate was centrifuged at 10,000 g for 10 min at 4°C and the supernatant was collected and diluted 10 times before assaying for triglycerides. The procedure followed was according to the manufacturers protocol.

### Oil-red-O staining

To examine lipid deposition in livers from experimental animals, 10 μm thick liver sections were subjected to Oil Red O staining using the NovaUltra (Woodstock, MD) Oil Red O staining kit. Briefly, frozen liver sections were air dried and fixed in formalin and washed under running water for 1–10 min. After rinsing with 60% isopropanol these sections were stained with freshly prepared Oil red O solution for 15 min. After rinsing again with 60% isopropanol and distilled water, the slides were mounted in mounting media and scanned using a Hamamatsu Nanozoomer Digital Pathology system (Hamamatsu City, Japan).

### Picrosirius staining

In order to evaluate hepatic collagen deposition, liver sections were stained using the Picrosirius Red staining kit (Polyscience, Warrington, PA). Briefly, liver sections were deparaffinized and hydrated with distilled water. Then the samples were immersed into solution A (Phosphomolybdic acid) for 2 min and rinsed with distilled water. Subsequently, the slides were kept in solution B (Picrosirius red stain) for 60 min and then in solution C (0.1 N hydrochloric acid) for 2 min. Following this the slides were placed in 70% ethanol for 45 s, again dehydrated, and used for mounting. The stained slides were scanned using Hamamatsu Nanozoomer Digital Pathology system (Hamamatsu City, Japan) and the digital information were stored for analysis.

### Metabolomics

Both initial and final serum samples and liver samples from all 3 experiments were subjected to metabolomic analysis by Dr. Shawn Campagna, the Director of the Biological and Small Molecule Mass Spectrometry Core facility at the University of Tennessee (https://chem.utk.edu/facilities/biological-and-small-molecule-mass-spectrometry-core-bsmmsc/).

### Statistical analysis

For the analysis of variables measured at the end of each experiment, one-way (for diet only) or two-way analysis of variance for multiple comparisons was performed with diet and sitagliptin treatment as main effects followed by post-hoc analysis using Tukey correction for multiple comparisons. Data are presented as mean +/− SEM. *P* values of 0.05 or less were considered as statistically significant.

## Results

### The effects of atherogenic diets and sitagliptin on hepatic oxidative stress markers

**Fig. 1:**
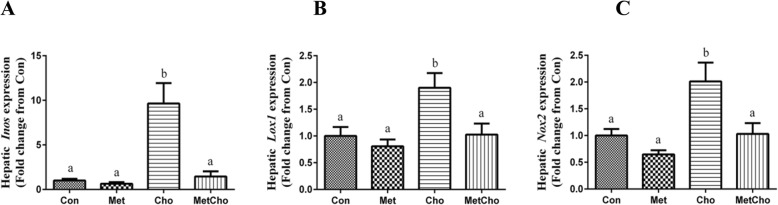
Expression analysis of hepatic oxidative stress marker genes in rats fed atherogenic diets. Sprague-Dawley rats were fed Con, high Met, high Cho, or high Met + high Cho diets ad libitum for 35 days, and gene expression of hepatic oxidative stress markers, *Inos* (**a**), *Lox1* (**b**) and *Nox2* (**c**) was measured. Data is represented as the mean ± SEM (*n* = 7 per group) and means annotated with different letters differ at *p* < 0.05

Diet-induced oxidative stress is an important and central mechanism in the development of NAFLD/NASH. In order to assess the impact of dietary Met, Cho and their combination on hepatic markers of oxidative stress, expression of *Nox2*, *Lox1*, and *Inos* mRNAs were measured in the liver (Experiment 1). We found that the high Met diet had no effect on expression of any of these markers compared to controls (Fig. 1a–c). On the contrary, the high Cho diet produced a significant 2-fold increase in expression of *Lox1* and *Nox2* (Fig. 1b, c). Additionally, the high Cho diet produced a 10-fold increase in *Inos* mRNA compared to controls (Fig. 1a). However, inclusion of Met in the high Cho diet (MetCho) reversed the stimulatory effect of Cho on expression of *Inos* (Fig. 1a), *Lox1* (Fig. 1b) and *Nox2* (Fig. 1c) mRNAs.

**Fig. 2:**
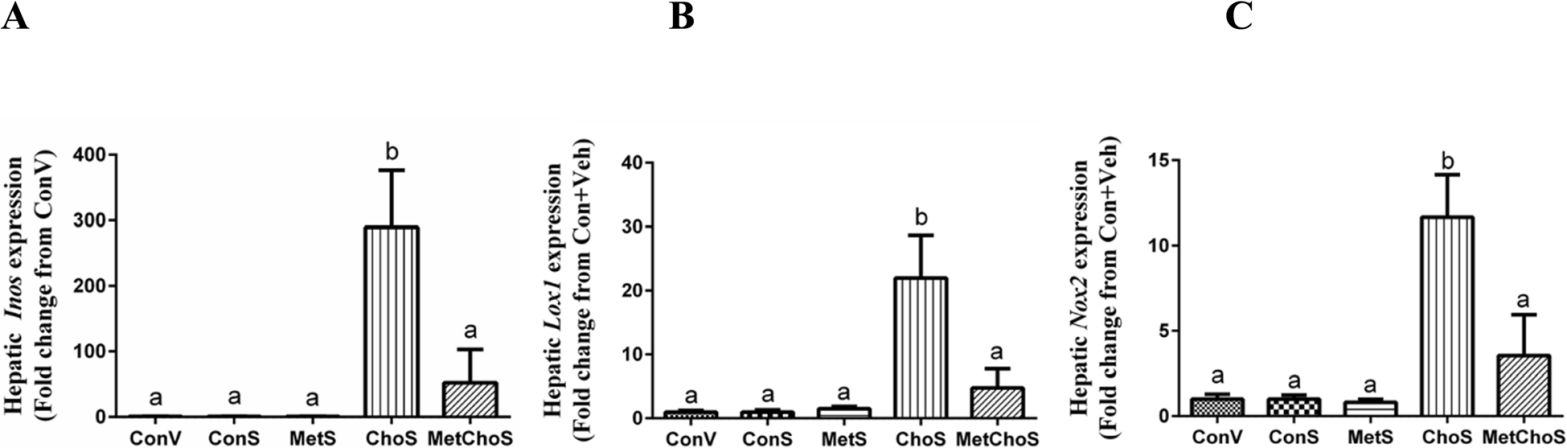
Expression analysis of hepatic oxidative stress marker genes in rats fed atherogenic diets and gavaged with sitagliptin. SD rats were fed Con or Met or Cho or MetCho diets ad libitum. All animals in the Met, Cho, and MetCho groups were orally gavaged with an aqueous suspension of sitagliptin (100 mg/kg/day) from day 10 through day 35, while half of the rats in the Con group were gavaged with vehicle and half the animals were gavaged with sitagliptin. At the end of the 5 weeks, livers were harvested and gene expression of hepatic oxidative stress markers, *Inos* (**a**), *Lox1* (**b**) and *Nox2* (**c**) was measured. Data are represented as the mean ± SEM (*n* = 7 per group) and means annotated with different letters differ at *p* < 0.05

To test whether sitagliptin would bring down the mRNA levels of oxidative stress markers which were increased by feeding the high Cho diet, sitagliptin was administered by oral gavage to rats on each diet starting from day 10 and continuing till the end of the experiment (35 days). Sitagliptin was without effect on hepatic *Inos, Lox1*, and *Nox2* mRNA expression in rats on the Con diet and it did not affect levels of these genes in rats on the Met diet (Fig. 2a–c). However, sitagliptin produced large increases in *Inos (*290-fold), *Lox1*(22-fold) and *Nox2* (12-fold) mRNA expression in livers from rats on the high Cho diet relative to controls (Fig. 2a–c). In contrast, the addition of Met to the high Cho diet reversed the stimulatory effect of sitagliptin on expression levels of these genes (Fig. 2a–c). This effect was also seen in the first experiment where Met supplementation reduced the increased expression of the oxidative stress markers observed in rats given sitagliptin and fed the high Cho diet. Importantly, diet alone or combined with sitagliptin had no significant effect on body composition among different groups (Additional file 1: Figure S1 and Additional file 2: Figure S2).

**Fig. 3:**
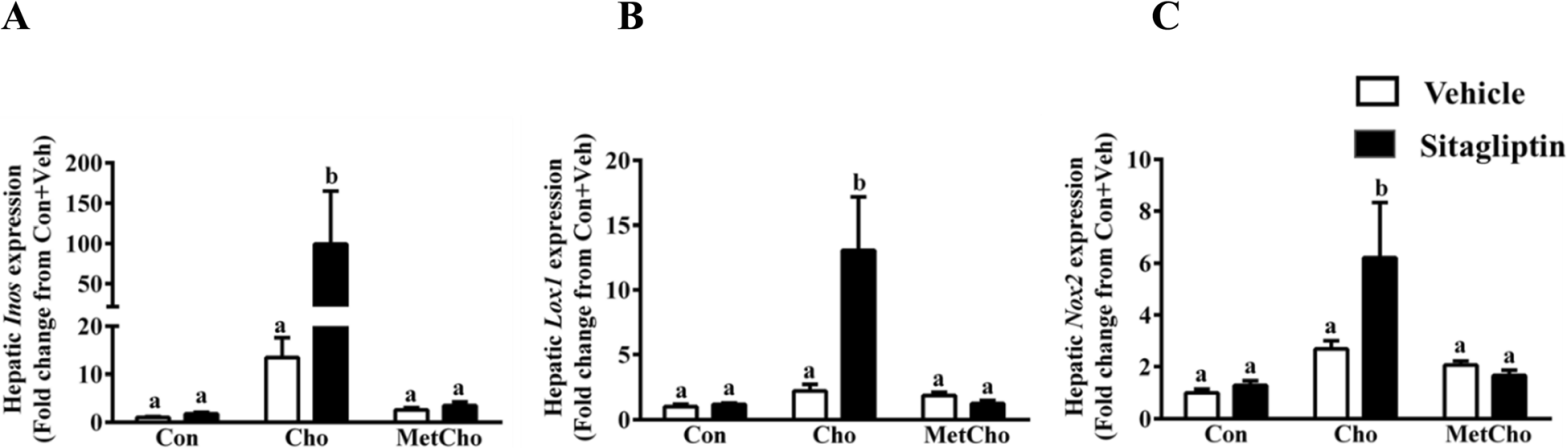
Sitagliptin exacerbates expression of hepatic oxidative stress marker genes in rats fed a high Cho diet. SD rats were fed Con, Cho, or MetCho diets ad libitum. As described in methods, from day 10 to 35, 50% of rats in each dietary group were gavaged with either vehicle or an aqueous suspension of sitagliptin (100mg/kg/day) and gene expression of *Inos* (**a**), *Lox1* (**b**) and *Nox2* (**c**) were measured. Data are represented as the mean ± SEM (*n* = 7–8 per group) and means annotated with different letters differ at *p* < 0.05

To directly test the hypothesis that sitagliptin amplified the high Cho-induced oxidative stress responses, a third experiment was conducted using vehicle- and sitagliptin-gavaged cohorts of rats with each diet (Experiment 3). As noted in Experiment 2, sitagliptin was without effect on hepatic oxidative stress markers in rats on the Con diet (Fig. 3a–c). The high Cho diet alone caused mild but non-significant increases in marker genes of oxidative stress (*Inos, Lox1, Nox2*). However, these genes were increased multifold when sitagliptin was combined with high Cho diet. The associated increases in expression of *Inos, Lox1, Nox2* were 100-, 13- and 6-fold, respectively (Fig. 3a–c). Inclusion of Met in the high Cho diet fully reversed the increased expression of oxidative stress markers produced in both the absence and presence of sitagliptin (Fig. 3a–c).

**Fig. 4:**
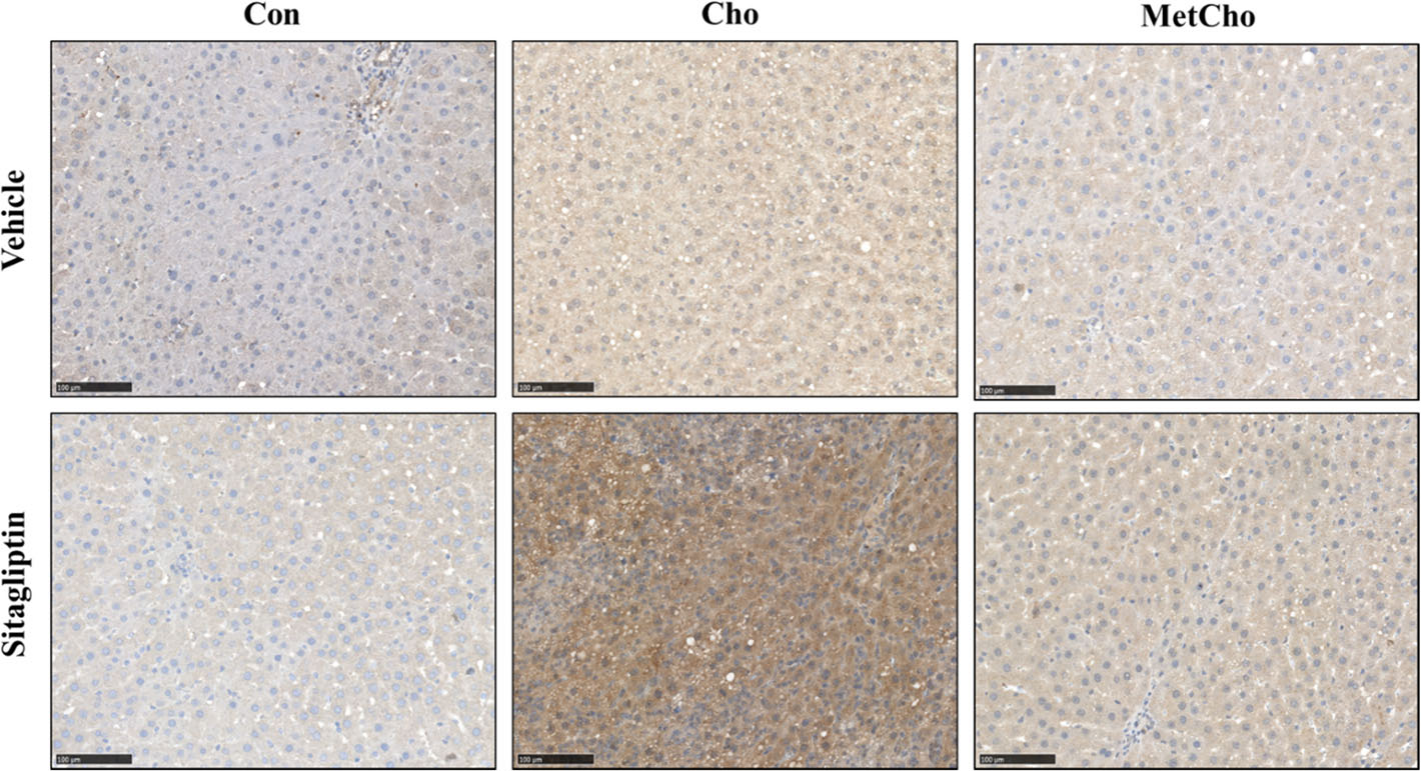
Sitagliptin exacerbates hepatic oxidative stress in rats fed a high Cho diet. SD rats were fed Con, Cho, or MetCho diets ad libitum. As described in methods, from day 10 to 35, 50% of rats in each dietary group were gavaged with either vehicle or an aqueous suspension of sitagliptin (100mg/kg/day). Representative IHC images of rat liver sections using anti-4 HNE antibody where 4-HNE adducts indicative of oxidative stress are evident in high Cho group gavaged with sitagliptin (3D). Scale bars = 100 μm

The immunohistochemistry of 4-hydroxynonenal (4-HNE), a product of lipid peroxidation and marker of oxidative stress was performed in liver (Fig. 4). As seen in Fig. 4, positive intense immunostaining of 4-HNE was evident in rats fed high Cho and given sitagliptin and reduced by addition of Met in high Cho diet (Fig. 4). This further confirmed the previous hypothesis that sitagliptin exacerbated the oxidative stress in a background of high Cho which was attenuated by Met.

### The effects of atherogenic diets and sitagliptin on hepatic steatosis

**Fig. 5:**
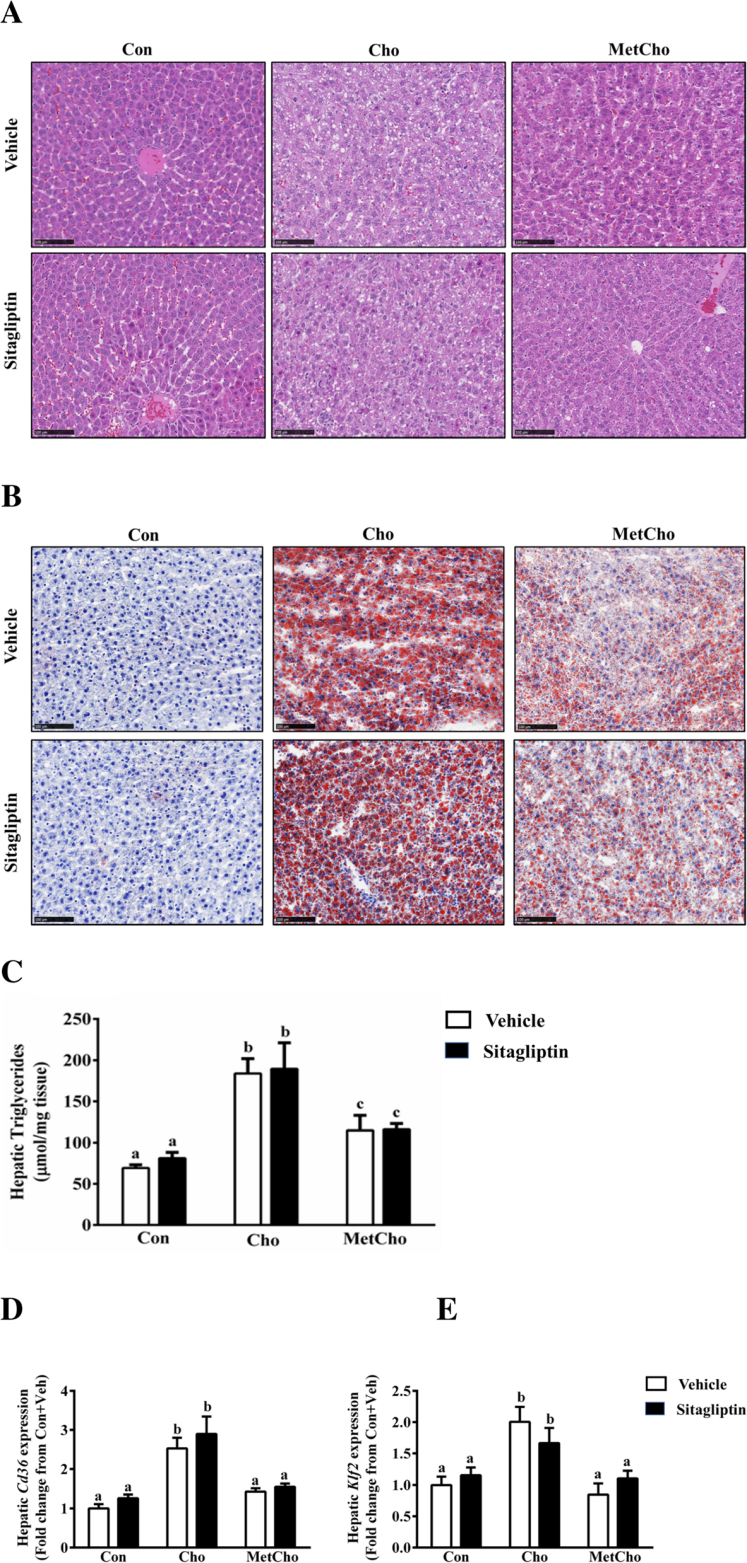
Effect of sitagliptin on hepatic steatosis in rats fed atherogenic diets. SD rats were fed Con or Cho or MetCho diets ad libitum. As described in methods, from day 10 to 35, 50% of rats in each dietary group were gavaged with either vehicle or an aqueous suspension of sitagliptin (100mg/kg/day). Sections from the largest lobe of the liver from each animal was used for H&E staining. Liver morphology of H&E stained liver sections from each group is represented (5A). Rats fed Cho and gavaged with vehicle or sitagliptin developed significant steatosis which was reduced by Met (**a**). Corresponding frozen liver sections were stained with Oil red O and maximum lipid deposition was observed in livers of rats fed high Cho followed by MetCho group. Sitagliptin was without effect on lipid deposition (**b**). Scale bars = 100 μm. Commercial ELISA kit was used for determination of triglyceride content in the liver of each rat group (**c**). Hepatic gene expression of *Cd36* (**d**) and *Klf2* (**e**) were measured. Data are represented as the mean ± SEM (*n* = 7–8 per group) and means annotated with different letters differ at *p* < 0.05

Because oxidative stress is known to induce lipid accumulation, we first examined the H&E-stained liver sections for changes in the number of lipid droplets. Liver sections from rats fed high Cho indicated increased lipid droplets which were reduced by addition of Met (Additional file 3: Figure S3, Fig. 5a). Further, sitagliptin did not impact the accumulation of lipid droplets in any of the dietary groups (Additional file 4: Figure S4, Fig. 5a). These results were independently examined and supported by Oil Red O staining (Fig. 5b). Additionally, hepatic triglyceride measurements showed that the high Cho diet increased lipid levels, and that sitagliptin had no modulatory effect in any of the diet groups (Fig. 5c). The addition of Met to the high Cho diet reversed part of the increase produced by high Cho alone, but liver triglyceride in the MetCho group was still higher than the Con group (Fig. 5c). Since hepatic lipid accumulation was increased in the high Cho group and lowered by inclusion of Met, we assessed the mRNA expression of *Cd36* (fatty acid translocase) and its transcriptional regulator, *Klf2* in liver tissues (Fig. 5d, e). These genes may contribute to hepatic lipid accumulation and were found to be increased in high Cho group compared to controls. Further, the addition of Met to the high Cho diet significantly lowered the expression of these genes. Sitagliptin had no effect on the expression of these genes in any of the dietary groups (Fig. 5d, e).

### The effects of atherogenic diets and sitagliptin on hepatic fibrosis

**Fig. 6:**
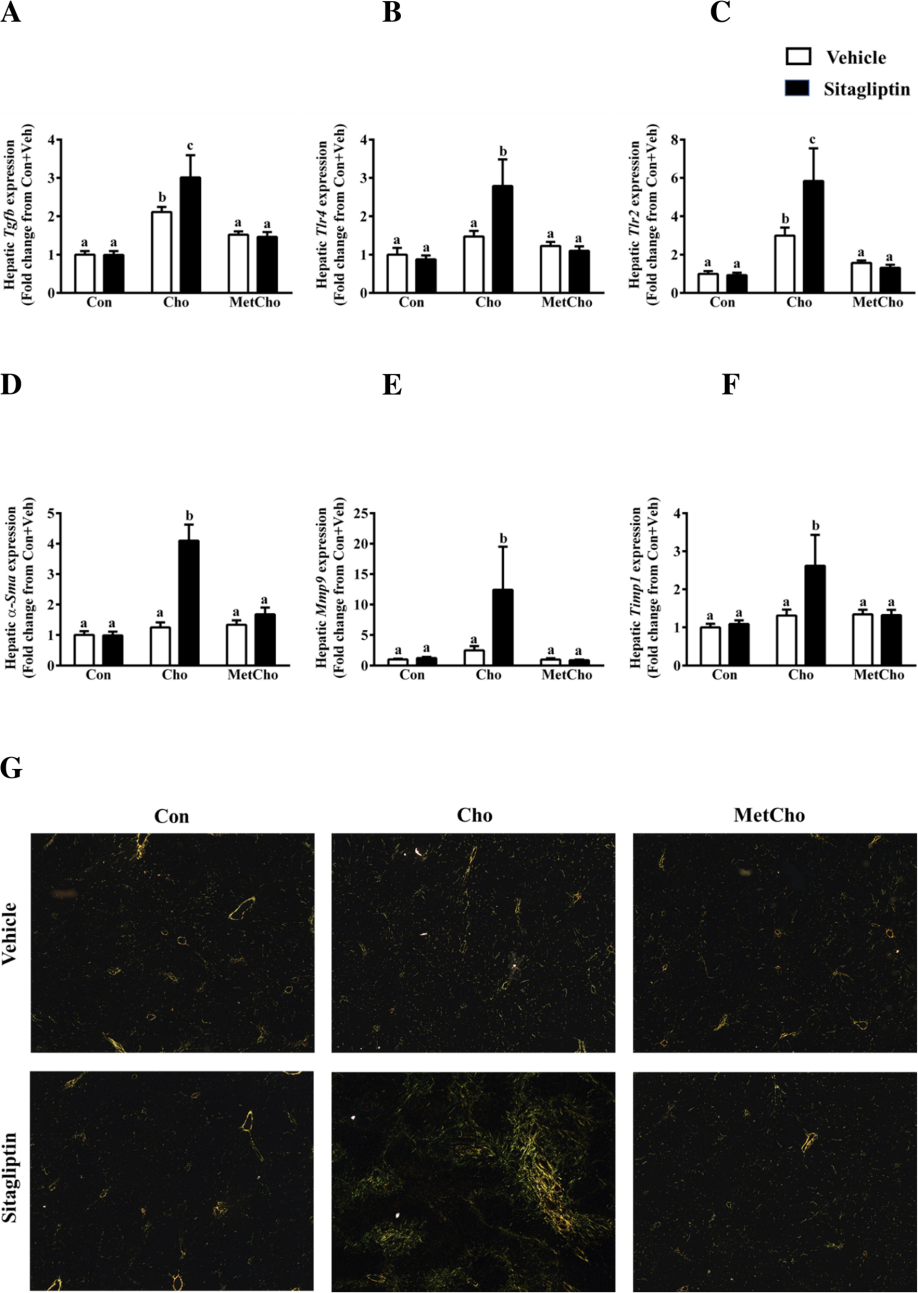
Effect of sitagliptin on liver fibrosis in rats fed atherogenic diets. SD rats were fed Con or high Cho diets ad libitum. As described in methods, from day 10 to 35, 50% of rats in each dietary group were gavaged with either vehicle or an aqueous suspension of sitagliptin (100mg/kg/day). Hepatic gene expression of *Tgfb* (**a**)*, Tlr4* (**b**), *Tlr2* (**c**), *a-sma* (**d**), *Mmp9* (**e**) and *Timp1* (**f**) were measured. Data are represented as the mean ± SEM (*n* = 7–8 per group) and means annotated with different letters differ at *p* < 0.05. Rat liver sections stained with picrosirius red were observed under polarized light microscope (**g**). Rats on high Cho and gavaged with sitagliptin exhibited dense collagen fibers (yellow colored). A profound decline in the collagen content was demonstrated in MetCho group as compared to Cho + Sitagliptin group (**g**)

Due to the strong associations among fibrosis, oxidative stress, and lipid accumulation, we investigated the fibrotic response in the context of the previously documented diet x drug interaction. Consistent with the previous reports of hepatic pathology in high Cho fed animals, fibrotic responses were marginally increased in high Cho diet fed animals compared to Con. Similar to oxidative stress responses, administration of sitagliptin exacerbated the fibrotic responses in the Cho group. Livers from the Cho + sitagliptin group had increased expression of mRNA for *Tgfb* (3-fold*), Tlr4* (2.8-fold), *Tll2* (5.8-fold), *a-Sma* (4-fold), *Mmp9* (12.4 fold), *and Timp1* (2.6 fold). As noted with other responses, the expression of these genes was suppressed by addition of Met to the Cho diet, implying a countering response by Met in hypercholesterolemic rats (Fig. 6a–f). Because we saw high expression of *Tgfb* and *a-Sma,* we further analyzed the deposition of fibrillar collagen by Picrosirius red staining and found increased collagen deposition in the Cho + sitagliptin group, but this increase was reduced by the addition of Met (Fig. 6g). These findings demonstrated that sitagliptin caused a highly significant increase of fibrotic responses in the rats fed high Cho diet which was countered by the addition of Met to the high Cho diet.

## Discussion

The current investigation of feeding a high Cho diet and its combination with high Met and intervention with sitagliptin yielded two important findings, a) a phenomenal increase in oxidative stress and fibrosis in liver by sitagliptin in a high Cho background and b) protection from hepatic damage due to high Cho plus sitagliptin by the addition of Met.

Oxidative stress is one of the most important factors leading to hepatic injury in NAFLD. It plays a major role in the progression of simple steatosis to NASH. Further, the augmented generation of reactive oxygen species (ROS) induces lipid peroxidation leading to inflammation and fibrogenesis by the activation of stellate cells [36–38]. Both Cho and Hcy (formed from Met) are associated with increasing oxidative stress [9, 22]. Most Western diets are comprised of animal products like meat, dairy and poultry, and these are rich in both Cho and Met. Although Hcy/Met and Cho have been independently associated with NAFLD, the combined effects of Met and Cho in the context of NAFLD is not well studied. Therefore, we investigated the effects of feeding a combination of high Met and high Cho with the objective of examining if there would be an additive effect of Met plus Cho on markers of oxidative stress in the liver (Experiment 1). Since oxidative stress plays an important role in the pathophysiology of NAFLD/NASH and oxidized LDL, inducible nitric oxide synthase and NADPH generated oxidative stress have been associated with the severity of NAFLD [39–43], we measured the mRNA expression of *Lox1*, *Inos* and *Nox2* as candidate biomarkers of oxidative stress. The rats fed high Met did not show an increase in hepatic markers of oxidative stress, showing expression levels that were comparable to animals on the Con diet. It was only in animals on the high Cho diet where we saw increased expression of markers of oxidative stress. However, we did not see an additional increase in the expression of oxidative stress markers in rats fed the combination diet (MetCho). Importantly, addition of Met to high Cho (MetCho) almost abolished the pathological changes that were seen with high Cho alone. The countering of oxidative stress by Met in high Cho fed rats was a serendipitous finding. Although there are studies documenting an association between toxic effects of higher intake of Met in liver due to Hcy (formed from Met), there are also reports on the hepatoprotective effects of Met. Studies on Met reducing sodium fluoride mediated hepatotoxicity [44] and the reduction of hepatotoxic effects of acetaminophen by incorporating Met with the drug are reported [30]. Additionally Met eliciting protection from nephrotoxicity [45], and reducing the severity of arthritis in rats [46] are some representative studies that support beneficial effects observed due to the incorporation of Met. On the lines of beneficial effects of Met and in the context of our work, it is relevant that there are several reports about the antioxidative effects of this amino acid [32, 45, 47].

Using the same dietary approach, we investigated the role of sitagliptin in the next set of experiments. Sitagliptin, a dipeptidyl peptidase-4 (DPP-4) inhibitor is an antidiabetic drug with documented anti-oxidative and anti-inflammatory properties [33–35]. Independent of glucose lowering, the DPP-4 inhibitors have been shown to have other health benefits. Among them, sitagliptin is known to improve liver steatosis by inhibiting fatty acid synthesis in the liver [48, 49]. Further, sitagliptin was shown to ameliorate the oxidative stress responses in diabetic nephropathy [50]. The rationale for using a DPP-4 inhibitor in our studies was based on its lipid lowering and anti-oxidative properties, and also because many type 2 diabetics have lipid abnormalities like hypercholesterolemia and take this drug to manage their blood glucose. Therefore, we were interested in investigating if sitagliptin would alleviate the hepatic oxidative stress and liver injury resulting from feeding a high Cho diet (Experiment 2). Contrary to prediction, sitagliptin phenomenally increased hepatic markers of oxidative stress in rats fed a high Cho diet. Such effects were not seen in rats on high Met as in the previous experiment (Experiment 1), and importantly, sitagliptin was without any effect when combined with high Met. Most interestingly, inclusion of Met countered the oxidative stress responses that were exacerbated by sitagliptin in the high Cho group.

In our studies, we used sitagliptin to investigate if it elicited beneficial effects of reducing inflammatory and oxidative stress responses based on the literature reports. Sitagliptin is reported to cause delay in gastric emptying, increase in insulin sensitivity and produce anti-oxidative and anti-inflammatory effects (which are independent of hypoglycemic effects) [33, 34]. Therefore, the increase in hepatic oxidative stress by sitagliptin in rats fed the high Cho diet was unexpected and similar to our recent findings where sitagliptin increased the hepatic inflammation in hypercholesterolemic rats [51]. In support of our findings of the harmful effects of sitagliptin in high Cho fed animals, there are reports of sitagliptin causing cardiac [52], hepatic [53, 54] and pancreatic damage [55]. From our results, we were able to confirm that a) only rats on high Cho had increase in oxidative stress and lipid accumulation, b) oxidative stress responses in high Cho group were exacerbated by sitagliptin, and c) incorporation of Met in high Cho (MetCho) had a protective effect against oxidative stress mediated liver damage.

In view of the clinical importance of sitagliptin for managing hyperglycemia in type 2 diabetics, and also since hypercholesterolemia is commonly seen in type 2 diabetics, we conducted another experiment to better understand the effect of drug and diet interaction (Experiment 3). Since Met alone did not cause any significant changes, we stayed with only three dietary groups (e.g., Con, 2.0% Cho and 1.5% Met+ 2.0% Cho). We investigated the drug and diet effect by having both sitagliptin and vehicle groups for each diet. In addition to markers of oxidative stress, we also examined additional markers of hepatic damage such as fibrosis and factors that influence triglyceride accumulation and promote steatosis. This experiment clearly established a negative diet x drug interaction with the high Cho diet and sitagliptin that was not evident in the Con group and produced significant hepatic damage. In contrast, inclusion of Met in the high Cho diet ameliorated the harmful interaction between sitagliptin and high Cho, and reversed the elevated markers of oxidative stress and fibrosis. Also, as seen in the first and second experiments, we found increased lipid accumulation in the rats on high Cho that was unaffected by sitagliptin. Again, we found that Met lowered the Cho-induced hepatic lipid accumulation. We were also able to explicitly demonstrate an increase in expression of *Cd36* gene, a fatty acid translocase (FAT) in the liver of rats fed the high Cho diet. In a recent study, it was demonstrated that *Cd36* directly contributes to the development of NAFLD under high free fatty acid condition via modulating their uptake in hepatocytes [56]. Further it is reported that deletion of *Cd36* improved hepatic steatosis, insulin sensitivity, and reduced systemic inflammation [56]. In addition to *Cd36,* we also examined the expression of *Klf2,* a positive regulator of the *Cd36* gene. *Klf2* is known to promote hepatic steatosis through upregulation of *Cd36* expression [57] and *Klf2* mRNA expression was increased in rats fed high Cho. We also analyzed the markers of fibrosis (*Tgf-b, Tlr-2, Tlr-4, a-Sma, Mmp9, Timp1*). As observed for oxidative stress, the expression of these fibrotic markers was significantly higher in the rats on high Cho diet administered with sitagliptin. *Tgf-b, Tlr-2* and *Tlr-4* activate pro-fibrogenic signaling pathways which play an important role in the development of NASH [58, 59]. *a-Sma* is a marker of activated hepatic stellate cells [60] that produce collagen fibers and increase the collagen deposition typically seen with fibrosis. In addition to *a-Sma*, matrix metalloproteinases such as *Mmp9* and *Timp1* are also involved in the development and progression of liver inflammation and fibrosis [61]. Inclusion of Met elicited a beneficial effect in alleviating increased expression of these markers as evidenced in animals on the Met plus Cho diet.

In the present work, we demonstrated Met-mediated reduction of oxidative stress, lipid accumulation, and fibrosis in livers of rats on the high Cho diet and administered with sitagliptin. Although there are studies documenting dietary restriction of Met increasing longevity, insulin sensitization, reduction of adiposity in addition to increase in both energy intake and expenditure [26–28], there are not many studies of high Met-mediated protection/prevention of hepatic damage. Infact, there are studies documenting hepato-damaging effects of high dietary Met alone, perhaps secondary to HHcy from high Met [14]. Interestingly, HHcy also occurs with Met restriction [62, 63], but in this case there is no association between HHcy and atherosclerosis or atherogenic effects [64]. It is documented that S-adenosyl methionine (SAM), an intermediate in the transmethylation pathway, serves as a precursor for glutathione and thus provides protection against oxidative stress-induced liver injury [31]. Studies report an increase in the levels of SAM after feeding diets enriched with Met [65, 66]. After de-adenosylation, S-adenosyl homocysteine is converted to Hcy which can either be remethylated or routed towards the transsulfuration pathway. Intermediates of the transsulfuration pathway like cystathionine and taurine are documented antioxidants. Additionally, cysteine (cys) which is an intermediate in this pathway, serves as the precursor for the formation of glutathione which is the most potent biological antioxidant. Both cystine (the disulfide of cys) and cystathionine are also the sources for the formation of hydrogen sulfide, a gaseous molecule which has been shown to have anti-oxidative, vasodilatory and anti-inflammatory properties in addition to several other benefits [67, 68]. Most importantly, Met by itself has been shown to have anti-oxidative properties [32, 45, 47]. Analysis of the liver metabolomic profile in our studies also indicates an increase in the levels of cystine and cystathionine in rats fed high Met plus high Cho diet and these metabolites might be conferring protection against oxidative stress (data not shown). Additionally, serum metabolomic profile reported a decrease in the purine metabolites specifically inosine and guanosine in the rats fed high Cho diet and gavaged with sitagliptin (Additional file 5: Figure S5). These purine metabolites have documented antioxidative properties [69] and hence reduction in their levels in high Cho plus sitagliptin group might be the reason for increased oxidative stress responses seen in these animals. The protection against hepatic oxidative stress, lipid accumulation and fibrosis by Met is very interesting. It is plausible that Met and intermediates formed during its metabolism such as SAM or those formed during the transsulfuration pathway or hydrogen sulfide may act either individually or in concert to confer hepatoprotective effects but this question needs further exploration.

## Conclusion

Our data indicate that sitagliptin in conjunction with high Cho diet intensified the oxidative damage resulting in NASH like symptoms. Surprisingly, the negative effects of high Cho and sitagliptin were at least partially attenuated by the inclusion of high Met in the diet.

## Supplementary information

**Additional file 1: Figure S1.** Body weight and body composition analysis of rats fed Con, Met, Cho and MetCho diets. Sprague-Dawley rats (age = 6 weeks) were fed control (Con), methionine supplemented (Met), high cholesterol (Cho), or high methionine + cholesterol (MetCho) diets ad libitum for 35 days and body weight and body composition (by TD-NMR) was measured once in a week.

**Additional file 2: Figure S2.** Body weight and body composition analysis of rats fed Con, Met Cho and MetCho diets and treated with sitagliptin. Sprague-Dawley rats (age = 6 weeks) were fed control (Con), methionine-supplemented (Met), high cholesterol (Cho), or high methionine + cholesterol (MetCho) diets ad libitum for 35 days. From day 10 through day 35 animals of each group were orally gavaged with an aqueous suspension of sitagliptin (100 mg/kg/day). In addition of Con+sitagliptin, we had an additional Con and rats in this group were administered the vehicle (water); vehicle Con. Body weight and body composition (by TD-NMR) was measured once in a week.

**Additional file 3: Figure S3.** Representative H&E stained images of the rat livers fed Con, Met, Cho and MetCho diets. Hepatic lipid accumulation was seen in rats of high Cho (shown by arrows). This was significantly reduced in the MetCho group. Scale bars = 100 μm.

**Additional file 4: Figure S4.** Representative H&E stained images of the rat livers fed Con, Met, Cho and MetCho diets and gavaged with sitagliptin. Compared to controls, hepatic lipid accumulation is evident in rats fed high Cho diet and gavaged with sitagliptin. This was significantly reduced by addition of Met in high Cho diet. Scale bars = 100 μm.

**Additional file 5: Figure S5.** Effects of sitagliptin and atherogenic diets on purine metabolites levels. SD rats were fed ad libitum Con or Cho or MetCho diets for 35 days. From day 10 through day 35, half animals of each group were orally gavaged with vehicle and the remaining half with an aqueous suspension of sitagliptin (100 mg/kg/day). Terminal serum samples were collected and processed for detection of serum metabolites by LC-MS. T-test analysis was performed to show the differences within a group and a heat map was generated. Purine metabolites (inosine and guanosine) were reduced when sitagliptin was given to rats fed high Cho diet and have been highlighted.

## Data Availability

The datasets used and/or analysed during the current study are available from the corresponding author on reasonable request.

## Abbreviations

Cho: Cholesterol
DPP-4: Dipeptidyl peptidase-4
GLP-1: Glucagon like peptide 1
Hcy: Homocysteine
HHcy: Hyperhomocysteinemia
HTL: Homocysteine thiolactone
Met: Methionine
NAFLD: Nonalcoholic fatty liver disease
NASH: Nonalcoholic steatohepatitis
ROS: Reactive oxygen species
SAH: S-adenosylhomocysteine
SAM: S-adenosyl methionine
