# Supplementary material for: High levels of dietary methionine improves sitagliptin-induced hepatotoxicity by attenuating oxidative stress in hypercholesterolemic rats
Source: Nutr Metab (Lond). 2020 Jan 6;17:2. doi: 10.1186/s12986-019-0422-z (PMC6945706; doi:10.1186/s12986-019-0422-z)
Supplement: Supplementary file 1 — Additional file 1: Figure S1. Body weight and body composition analysis of rats fed Con, Met, Cho and MetCho diets. Sprague-Dawley rats (age = 6 weeks) were fed control (Con), methionine supplemented (Met), high cholesterol (Cho), or high methionine + cholesterol (MetCho) diets ad libitum for 35 days and body weight and body composition (by TD-NMR) was measured once in a week. [file 12986_2019_422_MOESM1_ESM.docx]

**Additional file 1**

**
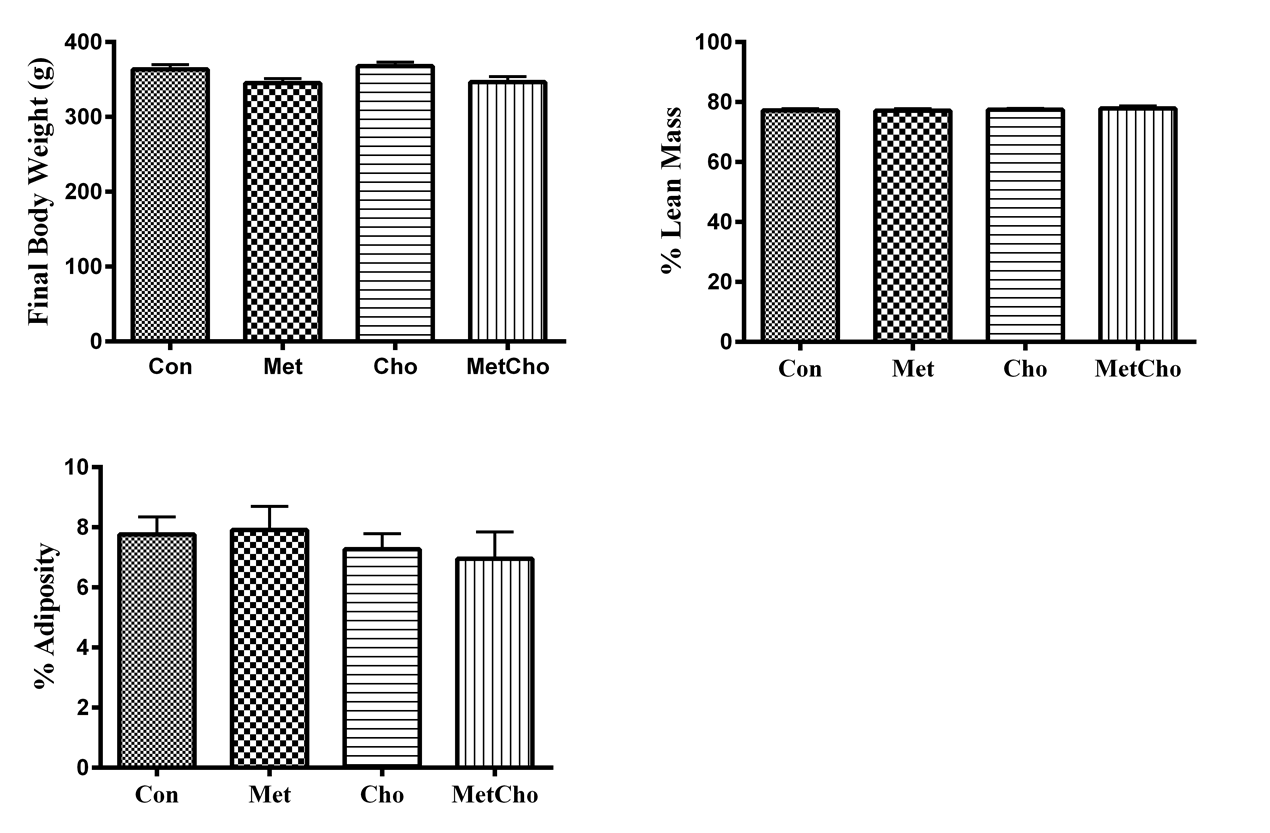
**

**Figure S1. Body weight and body composition analysis of rats fed Con, Met, Cho and MetCho diets.** Sprague-Dawley rats (age = 6 weeks) were fed control (Con), methionine supplemented (Met), high cholesterol (Cho), or high methionine + cholesterol (MetCho) diets ad libitum for 35 days and body weight and body composition (by TD-NMR) was measured once in a week.
