# Supplementary material for: High levels of dietary methionine improves sitagliptin-induced hepatotoxicity by attenuating oxidative stress in hypercholesterolemic rats
Source: Nutr Metab (Lond). 2020 Jan 6;17:2. doi: 10.1186/s12986-019-0422-z (PMC6945706; doi:10.1186/s12986-019-0422-z)
Supplement: Supplementary file 2 — Additional file 2: Figure S2. Body weight and body composition analysis of rats fed Con, Met Cho and MetCho diets and treated with sitagliptin. Sprague-Dawley rats (age = 6 weeks) were fed control (Con), methionine-supplemented (Met), high cholesterol (Cho), or high methionine + cholesterol (MetCho) diets ad libitum for 35 days. From day 10 through day 35 animals of each group were orally gavaged with an aqueous suspension of sitagliptin (100 mg/kg/day). In addition of Con+sitagliptin, we had an additional Con and rats in this group were administered the vehicle (water); vehicle Con. Body weight and body composition (by TD-NMR) was measured once in a week. [file 12986_2019_422_MOESM2_ESM.docx]

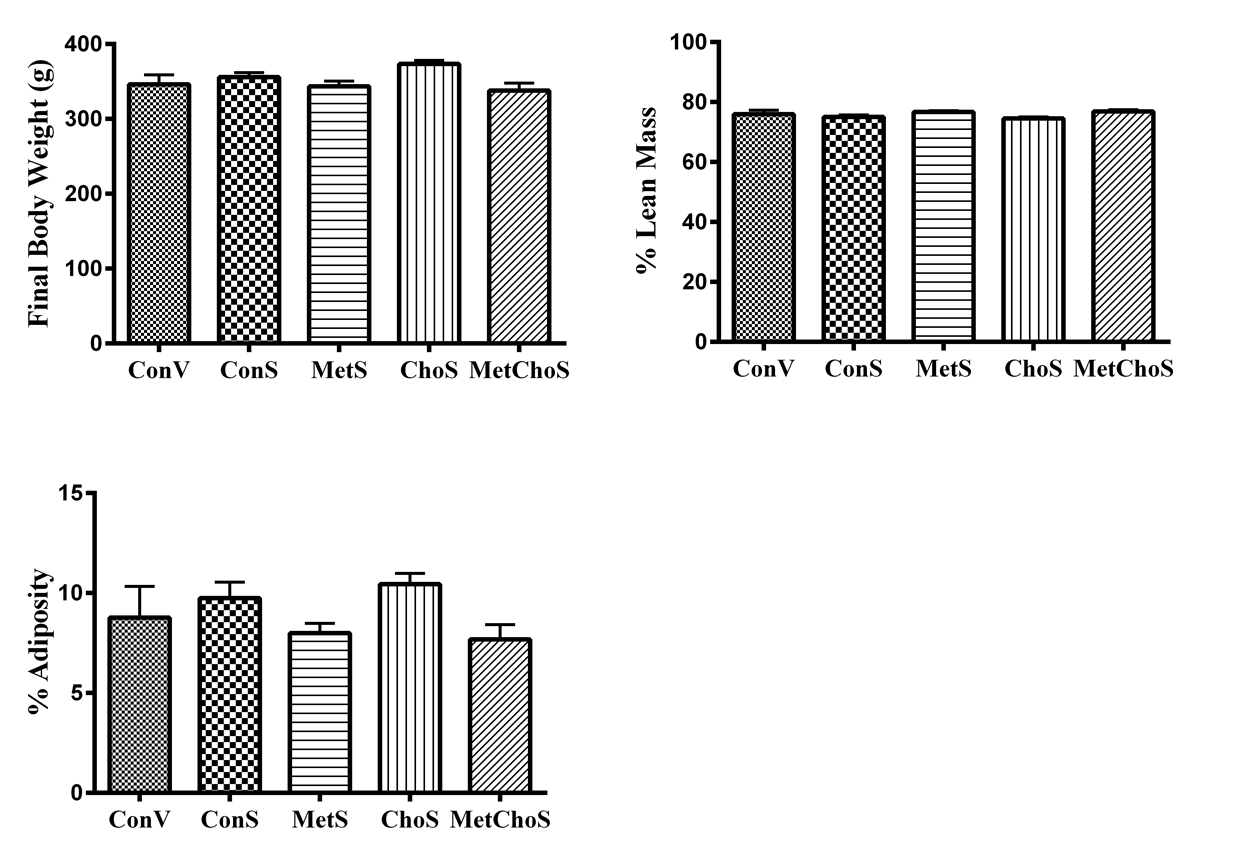


**Figure S2. Body weight and body composition analysis of rats fed Con, Met Cho and MetCho diets and treated with sitagliptin.** Sprague-Dawley rats (age = 6 weeks) were fed control (Con), methionine-supplemented (Met), high cholesterol (Cho), or high methionine + cholesterol (MetCho) diets ad libitum for 35 days. From day 10 through day 35 animals of each group were orally gavaged with an aqueous suspension of sitagliptin (100 mg/kg/day). In addition of Con+sitagliptin, we had an additional Con and rats in this group were administered the vehicle (water); vehicle Con. Body weight and body composition (by TD-NMR) was measured once in a week.
