# Supplementary material for: High levels of dietary methionine improves sitagliptin-induced hepatotoxicity by attenuating oxidative stress in hypercholesterolemic rats
Source: Nutr Metab (Lond). 2020 Jan 6;17:2. doi: 10.1186/s12986-019-0422-z (PMC6945706; doi:10.1186/s12986-019-0422-z)
Supplement: Supplementary file 3 — Additional file 3: Figure S3. Representative H&E stained images of the rat livers fed Con, Met, Cho and MetCho diets. Hepatic lipid accumulation was seen in rats of high Cho (shown by arrows). This was significantly reduced in the MetCho group. Scale bars = 100 μm. [file 12986_2019_422_MOESM3_ESM.docx]

**
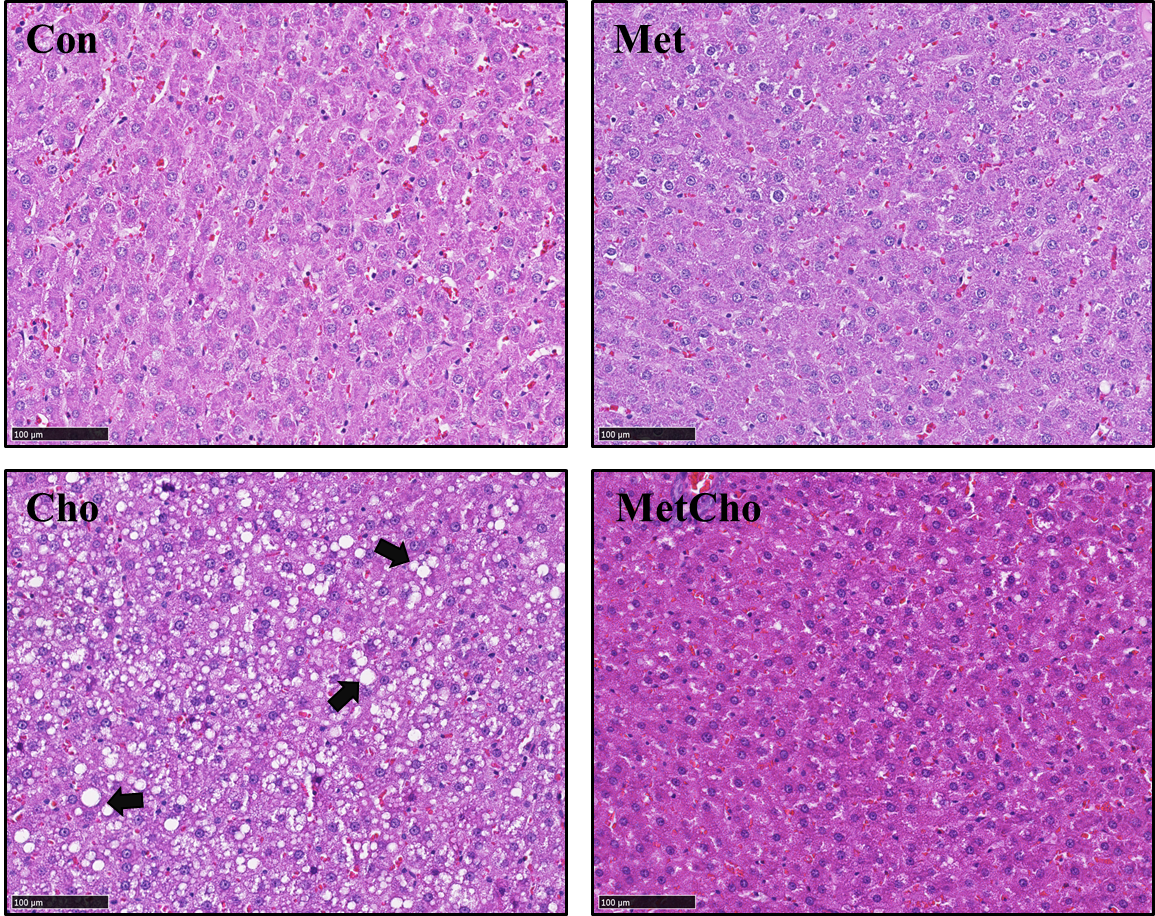
**

**Figure S3.** Representative H&E stained images of the rat livers fed Con (A), Met (B), Cho (C) and MetCho (D) diets. Hepatic lipid accumulation was seen in rats of high Cho (shown by arrows). This was significantly reduced in the MetCho group. Scale bars= 100µm.
