# Supplementary material for: High levels of dietary methionine improves sitagliptin-induced hepatotoxicity by attenuating oxidative stress in hypercholesterolemic rats
Source: Nutr Metab (Lond). 2020 Jan 6;17:2. doi: 10.1186/s12986-019-0422-z (PMC6945706; doi:10.1186/s12986-019-0422-z)
Supplement: Supplementary file 4 — Additional file 4: Figure S4. Representative H&E stained images of the rat livers fed Con, Met, Cho and MetCho diets and gavaged with sitagliptin. Compared to controls, hepatic lipid accumulation is evident in rats fed high Cho diet and gavaged with sitagliptin. This was significantly reduced by addition of Met in high Cho diet. Scale bars = 100 μm. [file 12986_2019_422_MOESM4_ESM.docx]

**
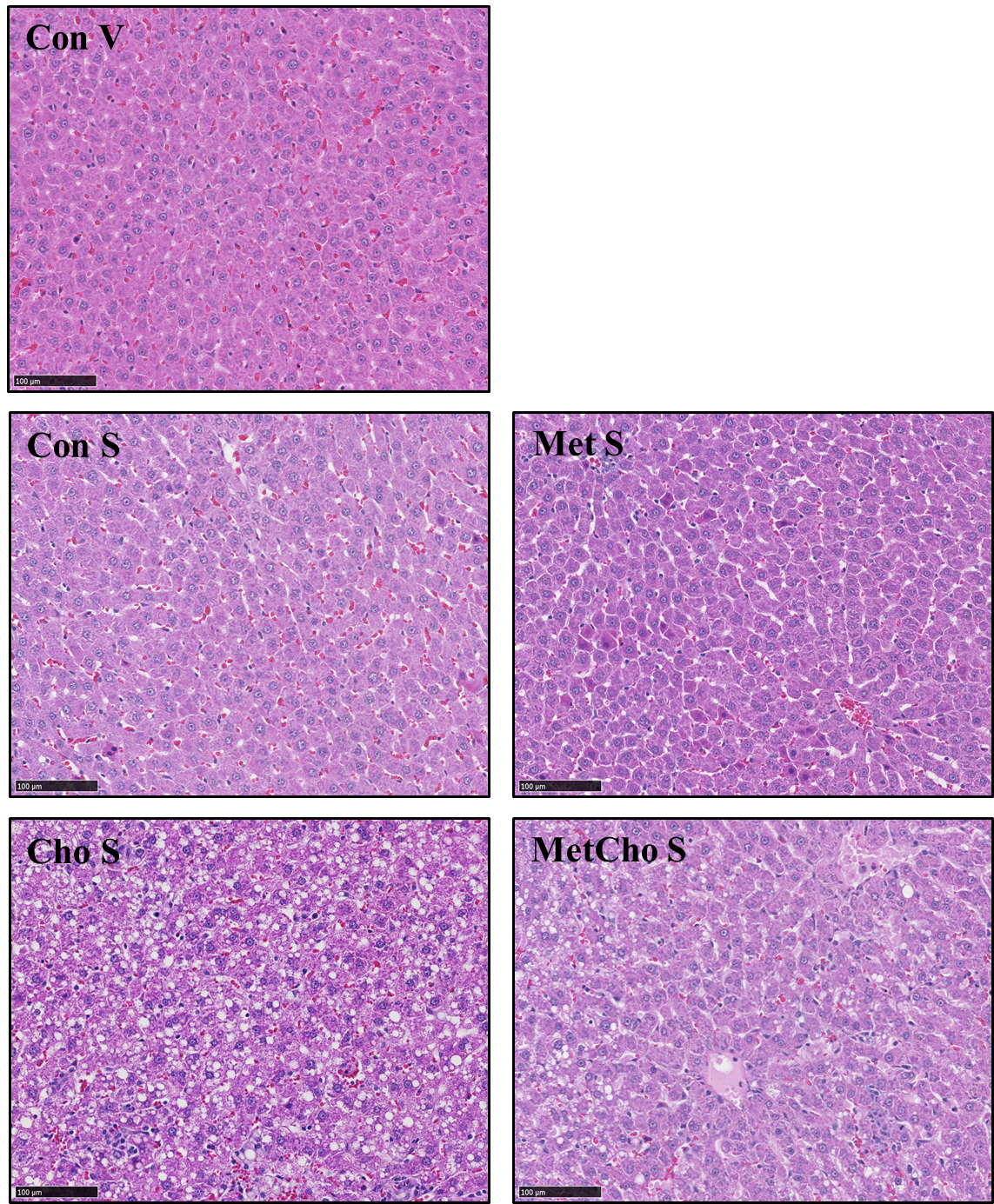
**

**Figure S4.** Representative H&E stained images of the rat livers fed Con (A), Met (B), Cho (C) and MetCho (D) diets and gavaged with sitagliptin. Compared to controls, hepatic lipid accumulation is evident in rats fed high Cho diet and gavaged with sitagliptin. This was significantly reduced by addition of Met in high Cho diet. Scale bars= 100µm.
