# Supplementary material for: High levels of dietary methionine improves sitagliptin-induced hepatotoxicity by attenuating oxidative stress in hypercholesterolemic rats
Source: Nutr Metab (Lond). 2020 Jan 6;17:2. doi: 10.1186/s12986-019-0422-z (PMC6945706; doi:10.1186/s12986-019-0422-z)
Supplement: Supplementary file 5 — Additional file 5: Figure S5. Effects of sitagliptin and atherogenic diets on purine metabolites levels. SD rats were fed ad libitum Con or Cho or MetCho diets for 35 days. From day 10 through day 35, half animals of each group were orally gavaged with vehicle and the remaining half with an aqueous suspension of sitagliptin (100 mg/kg/day). Terminal serum samples were collected and processed for detection of serum metabolites by LC-MS. T-test analysis was performed to show the differences within a group and a heat map was generated. Purine metabolites (inosine and guanosine) were reduced when sitagliptin was given to rats fed high Cho diet and have been highlighted. [file 12986_2019_422_MOESM5_ESM.docx]

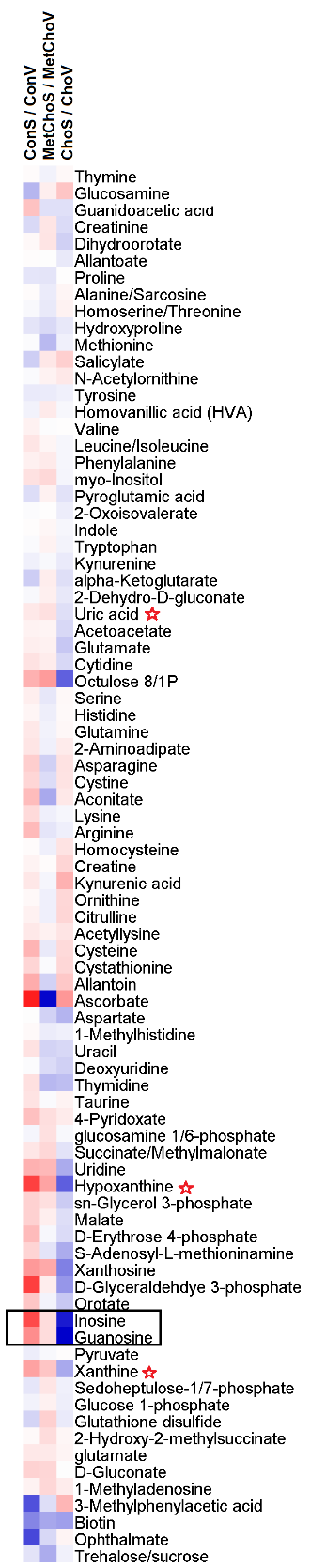

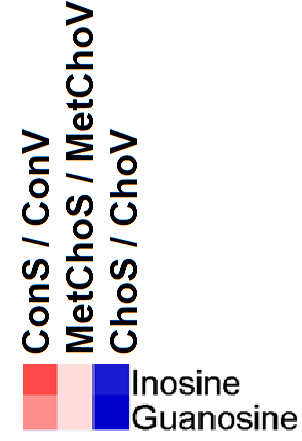

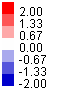


**Figure S5. Effects of sitagliptin and atherogenic diets on purine metabolites levels.** SD rats were fed *ad libitum* Con or Cho or MetCho diets for 35 days. From day 10 through day 35, half animals of each group were orally gavaged with vehicle and the remaining half with an aqueous suspension of sitagliptin (100 mg/kg/day). Terminal serum samples were collected and processed for detection of serum metabolites by LC-MS. T-test analysis was performed to show the differences within a group and a heat map was generated. Purine metabolites (inosine and guanosine) were reduced when sitagliptin was given to rats fed high Cho diet and have been highlighted.
